# From Antisense RNA to RNA Modification: Therapeutic Potential of RNA-Based Technologies

**DOI:** 10.3390/biomedicines9050550

**Published:** 2021-05-14

**Authors:** Hironori Adachi, Martin Hengesbach, Yi-Tao Yu, Pedro Morais

**Affiliations:** 1 Center for RNA Biology, Department of Biochemistry and Biophysics, University of Rochester Medical Center, 601 Elmwood Avenue, Rochester, NY 14642, USA; hironori_adachi@urmc.rochester.edu; 2 Institute for Organic Chemistry and Chemical Biology, Johann Wolfgang Goethe-University Frankfurt, Max-von-Laue-Str. 7, 60438 Frankfurt, Germany; hengesbach@nmr.uni-frankfurt.de; 3 ProQR Therapeutics, Zernikedreef 9, 2333 CK Leiden, The Netherlands

**Keywords:** antisense technology, epitranscriptomics, RNA modification, ADAR, pseudouridylation, 2′-O-methylation, gapmers, siRNAs, splice-modulating oligonucleotides

## Abstract

Therapeutic oligonucleotides interact with a target RNA via Watson-Crick complementarity, affecting RNA-processing reactions such as mRNA degradation, pre-mRNA splicing, or mRNA translation. Since they were proposed decades ago, several have been approved for clinical use to correct genetic mutations. Three types of mechanisms of action (MoA) have emerged: RNase H-dependent degradation of mRNA directed by short chimeric antisense oligonucleotides (gapmers), correction of splicing defects via splice-modulation oligonucleotides, and interference of gene expression via short interfering RNAs (siRNAs). These antisense-based mechanisms can tackle several genetic disorders in a gene-specific manner, primarily by gene downregulation (gapmers and siRNAs) or splicing defects correction (exon-skipping oligos). Still, the challenge remains for the repair at the single-nucleotide level. The emerging field of epitranscriptomics and RNA modifications shows the enormous possibilities for recoding the transcriptome and repairing genetic mutations with high specificity while harnessing endogenously expressed RNA processing machinery. Some of these techniques have been proposed as alternatives to CRISPR-based technologies, where the exogenous gene-editing machinery needs to be delivered and expressed in the human cells to generate permanent (DNA) changes with unknown consequences. Here, we review the current FDA-approved antisense MoA (emphasizing some enabling technologies that contributed to their success) and three novel modalities based on post-transcriptional RNA modifications with therapeutic potential, including ADAR (Adenosine deaminases acting on RNA)-mediated RNA editing, targeted pseudouridylation, and 2′-O-methylation.

## 1. Introduction

mRNA processing reactions are critical in the pathway of gene expression. Pre-mRNA is synthesized in the nucleus, where it undergoes capping/polyadenylation and splicing, as well as several post-transcriptional modifications. The mRNA is then transported out of the nucleus to the cytoplasm, where it is translated into protein and subsequently degraded [1]. This whole process involves a highly regulated network of events that are all critical for the cell’s functioning. Many of the monogenic genetic disorders caused by DNA mutations in a single gene [2] often affect one or several processing events, usually resulting in the production of non-functional proteins and severe or even fatal disease phenotypes. For many decades, the strategy for the correction of these mutant proteins was to screen small molecules for “correctors” that could restore their function (thus, ameliorate disease phenotypes) or for inhibitors that would block their activity when it would be toxic for the cell [3]. However, some of the well-known limitations of some small molecule drugs is the lack of a clear mechanism of action (MoA) [4] and target specificity, potentially leading to toxic off-target effects [5]. This has prompted a search for macromolecules that would be highly specific and deliverable to the target tissues [6]. With their clear base-pairing hybridization rules, antisense oligonucleotides (AONs) were obvious candidates as an entirely new therapeutic technology to repair monogenic disorders’ underlying causes [7]. Antisense technology represented a complete change in the way drugs are discovered and developed up to the clinical level. The Watson-Crick hybridization properties of nucleic acids enabled a faster and rational design together with target-specificity unmatched by any small molecule.

Three main RNA processing MoA emerged that took antisense technologies to the clinical stage: (1) RNase H-dependent mRNA degradation with gapmers, (2) RNA interference for siRNA-mediated degradation of transcripts, and (3) splicing modulation via steric blocking AONs. These MoA generated several FDA-approved drugs in recent years [8,9] and have become established platform technologies. This paper will describe their main features and will emphasize several breakthroughs, which will potentially become enabling technologies for novel MoA.

The emerging field of epitranscriptomics has enabled the identification of more than 170 nucleotide RNA modifications [10], offering chemical diversity in the nucleobase or sugar moieties. Such modifications can confer distinct properties to the RNA by affecting RNA structure, RNA-RNA and RNA-protein interactions, and ultimately function. In particular, the discovery of several guide RNA-directed RNA editing/modification processes [11] and how they affect RNA function and gene expression [12] have drawn immense attention in the last decades [13]. Notably, a new class of RNA editing therapeutics based on induced RNA modifications for the repair of genetic mutations has been recognized [14], that is, to edit and rewrite the messenger RNA. These new technologies can harness endogenous editing machinery through sequence features, chemical modifications, or secondary structural elements designed to recruit editing proteins [15]. This has been possible due to the advances in structural biology that enable an understanding of the structures of RNA editing proteins and how they interact with target RNA [16,17,18], as well as the breakthroughs in the development of new oligonucleotide chemical modifications [19]. This paper will also describe the main features of three novel epitranscriptomic MoA with the potential to become new therapeutic modalities, thus expanding the current scope of genetic diseases that classical antisense technology cannot address.

## 2. Enabling Technologies for Oligonucleotide Therapeutics

We have come a long way to successfully translate the early discoveries in oligonucleotide therapeutics into proof-of-concept in human trials with a meaningful impact on patients’ lives. One of the biggest challenges has been the efficient delivery of oligonucleotides to the site of action (organ, tissue, cells, and subcellular localization depending on the MoA). Here, we summarize the leading platform technologies or administration procedures that enabled these macromolecules to reach the site of action and hybridize to their target RNA in a highly sequence-specific manner [20].

### 2.1. Chemical Modifications

**Figure 1:**
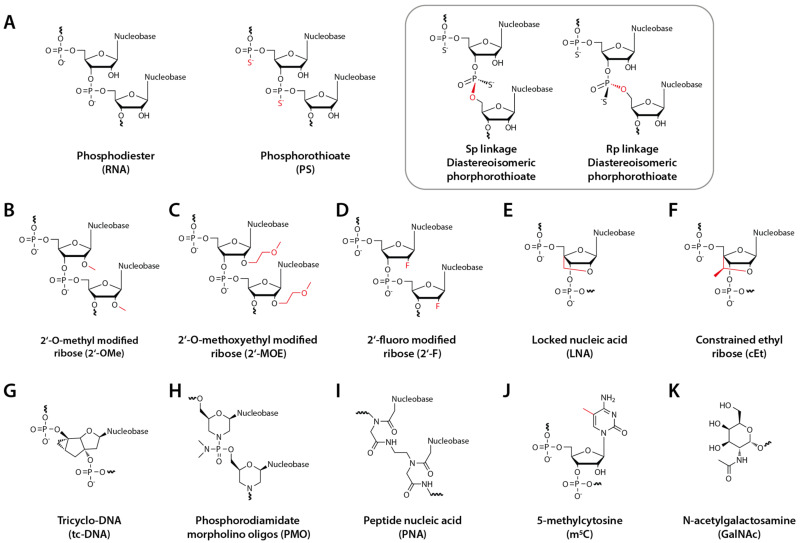
Structures of AON chemical modifications and the GalNAc conjugate. (**A**) Phosphodiester and Phosphorothioate (PS), inset: Rp and Sp diastereoisomers, (**B**) 2′-O-methyl modified ribose (2′-OMe), (**C**) 2′-O-methoxyethyl modified ribose (2′-MOE) (**D**) 2′fluoro (2′-F), (**E**) Locked nucleic acid (LNA) (**F**) Constrained ethyl (cEt), (**G**) Tricyclo-DNA (tcDNA), (**H**) Phosphorodiamidate morpholino oligos (PMO), (**I**) Peptide nucleic acid (PNA), (**J**) 5-methyl-cytosine (m^5^C), and (**K**) N-acetylgalactosamine (GalNAc).

Therapeutic oligonucleotides usually have chemical modifications in their phosphate backbone and sugar rings (Figure 1). The most common versions of AONs have phosphorothioate backbones (PS), where a sulfur atom replaces one oxygen atom in the phosphodiester group (Figure 1A). Besides, the 2′-OH group in the ribose sugar is replaced by a 2′-O-methyl group (2′-OMe) (Figure 1B). These two modifications were critical enabling technologies in the antisense technology field since they improve AONs’ therapeutic properties, in particular their stability and cellular uptake, while maintaining and even enhancing their affinity (via base-pairing) to the target sequences. More specifically, it has been reported that the PS-modification improves bioavailability and the cellular uptake properties of oligos [21] due to improved binding to serum protein. While PS has a minimal impact on target binding affinity compared to phosphodiester bonds, it increases resistance to nucleases [22]. Likewise, 2′-OMe also increases oligos’ stability [23] while favoring the A-form RNA configuration and thus increasing binding affinity to the target RNA [24]. Further, PS/2′-OMe oligos are easy and relatively cheap to synthesize. This allowed an influx of academic researchers and small biotech companies to screen therapeutic oligonucleotides with different MoA. Moreover, these modifications are compatible with a wide variety of therapeutic MoA. As such, up to this day, there are several clinical trials where the oligonucleotide drugs have these two modifications.

Due to the impact of chemical modifications in the properties of oligonucleotides, this field has witnessed a significant evolution in the last decades [19]. An entire portfolio of chemistries has been developed for a variety of ribose modifications, such as 2′-methoxyethyl (2′-MOE) [25], 2′-fluor (2′-F) [26], locked nucleic acid oligos (LNA) [27], constrained ethyl oligos (cEt) [28] and tricyclo-DNA oligos (tc-DNA) [29] (Figure 1G). Likewise, the chemical properties of phosphate backbone modifications, including phosphorodiamidate morpholino oligos (PMO) [30] and peptide-nucleic acid oligos (PNA) [31] (Figure 1H and Figure 1I, respectively), have been extensively studied. In some cases, nucleobase modifications, for example, 5-methylcytosine (m^5^C) [32] (Figure 1J) (for a comprehensive review: [33]), have drawn tremendous attention as well. Different chemical modifications can confer specific properties to oligonucleotides. For instance, PMO, PNA, 2′-F, m^5^C, 2′-OMe, 2′-MOE, and especially LNA increase binding affinity to the target [34]. The LNA modification, often used in situations where there is a need to increase the binding affinity to the target RNA, can favor an A-form duplex conformation with the target, thus increasing the melting temperature [35]. On the other hand, PNA, 2′-OMe, 2′-MOE, and particularly m^5^C (in CpG dinucleotide stretches) reduce the chances for oligo-induced innate immune responses [36]. Some modifications (PMO and PNA) have a neutral charge, conferring distinct properties to the oligonucleotides. This arsenal of chemical modifications allows adapting a particular oligonucleotide to a required mode of action and administration route.

It has been recently suggested that the PS backbone, which typically consists of a mixture of two different diastereoisomers, Rp or Sp (Figure 1A inset), could also be used in a stereodefined configuration to improve the oligonucleotide therapeutic properties [37]. However, there is still some debate around this hypothesis [38].

There is also a significant amount of work in understanding how the chemical modifications affect the oligonucleotides’ ability to bind to cellular proteins, affecting their pharmacokinetic properties and, ultimately, their efficacy [39].

### 2.2. Administration Routes

One of the biggest advantages of small molecules is the convenient way they can be administered to the body. For therapeutic oligonucleotides, there have been immense advances catalyzed by multiple clinical trials performed in various therapeutic areas. Before reaching the subcellular target site (which can vary according to the MoA), oligonucleotides need to reach the target tissue, either by systemic delivery or a local administration route [40]. Systemic delivery (intravenous or subcutaneous) can be challenging for several reasons. The oligonucleotides must be sufficiently stable (i.e., chemically modified) to resist degradation in the serum. Further, a certain fraction of the administered dose will be eliminated from the organism via excretion. For instance, if the target tissue is the central nervous system, the blood-brain barrier will prevent oligos from reaching it. While there are already a few therapeutic oligonucleotides systemically administered, local administration methods have attracted attention. Several examples have been described for local administration: intrathecal [41], intramuscular [42], intravitreal injection [43,44], topical [45], and nebulization/inhalation [46,47].

The intravitreal route is particularly promising. There is currently a high unmet need for treatment for inherited retinal diseases (IRDs), which cause blindness [48]. In 2017, an AAV-based gene therapy was approved for Leber’s congenital amaurosis (a rare disease that causes progressive blindness). Despite the good reception of the drug, the treatment involves general anesthesia and a small surgery with a complicated subretinal injection procedure which is not without risks, such as retinal detachment. A recent Phase I/II clinical trial (NCT03913143) has demonstrated the potential of intravitreous injection for delivery of an oligonucleotide (sepofarsen) for treatment of the same disease, although repairing a different target gene (CEP290 and the c.2991 + 1655A > G mutation) by restoring correct splicing [43]. In this study, which is currently on Phase II/III, the intravitreally injected splice-modulating oligo was well tolerated and improved visual acuity in patients [49]. This breakthrough shows the promise of oligos in eye disorders [50].

Among already FDA-approved oligo drugs, there are three that use the intravenous route (patisiran, eteplirsen, and golodirsen), three that are injected subcutaneously (mipomersen, inotersen, and givosiran), one that is injected intravitreally (fomivirsen, although it is not available anymore), and one that is injected intrathecally (nusinersen) [34].

### 2.3. Delivery Technologies

The delivery of oligonucleotide therapeutics is arguably the biggest challenge in this area. Before reaching their target site in the cells, oligonucleotides need to face several hurdles, such as the risk of RNase-mediated degradation and endosomal entrapment [51]. With the recent advances in novel delivery technologies for oligonucleotides, namely exosomes [52] (for a review see [53]) and adeno-associated viral vectors [54], two technologies have emerged with proven clinical results to overcome the delivery challenges of oligos: N-acetylgalactosamine-conjugates (GalNAc) and the use of lipid-nanoparticles (LNPs) (discussed below). A significant portion of oligonucleotide therapeutics currently in clinical trials uses one of these delivery modalities and targets liver diseases or genes mainly expressed in liver hepatocytes. Recently, however, an adeno-associated viral vector delivering an AON entered a clinical trial and will also be discussed below (Section 2.3.3).

#### 2.3.1. GalNAc-Conjugated Oligonucleotides

One of the most important technological leaps in oligonucleotide therapeutics was the finding that small chemical entities naturally recognized by cellular membrane receptors in liver cells (hepatocytes) could be attached to oligonucleotides (such as siRNAs) to improve their delivery, especially in the context of systemic administration. Hepatocytes naturally express a receptor, known as the asialoglycoprotein receptor (ASGPR), with a carbohydrate-binding protein (C-type lectin) that can bind and internalize glycoproteins with a GalNAc residue (Figure 1K). Hangeland et al. proposed the covalent attachment of GalNAc moieties to oligonucleotides to improve their tissue distribution properties in vivo [55], a concept further expanded by Prakash et al. with improvements in the chemistry of GalNAc (tri-antennary) [56]. GalNAc binds to asialoglycoproteins receptors in hepatocytes and facilitates therapeutic nucleic acids’ delivery to the liver [57]. While it has been reported that the ASGPR receptor can be detected in the surface of non-hepatic cells, specifically in activated T-cells [58], no GalNAc-AON-mediated off-target effects have been identified in these cells.

Some successful examples of GalNAc-RNA drugs will be discussed below in Section 3.2.

#### 2.3.2. Lipid-Nanoparticles Formulations (LNPs)

In recent years, there has been tremendous progress in the development of LNPs. These nanoparticles consist of amphipathic lipids that typically contain a hydrophilic head and a hydrophobic alkyl chain. Usually, the lipids are cationic or ionizable cationic to interact with (and thus carry) negatively charged therapeutic oligonucleotides, such as siRNA molecules. Zimmerman et al. showed that ionizable cationic LNPs (known as Stable Nucleic Acid-Lipid Particles, or SNALPs) could successfully deliver siRNAs systemically in non-human primates [59]. It appears that siRNA-LNP complexes significantly improve delivery in hepatocytes since they bind to Apolipoprotein E (ApoE), which can be incorporated in hepatocytes via an ApoE receptor present at the cellular membrane, thus favoring cellular uptake and escape from the cellular compartments known as endosomes. These synthetic particles can encapsulate nucleic acids and execute multiple functions: protecting the therapeutic nucleic acids from RNase degradation and improving their cellular uptake properties. Typically, oligonucleotides can be incorporated into SNALPs by mixing the nucleic acids with lipids in ethanol at low pH (4.0) [60]. The potential applications of LNPs as delivery vehicles have been extensively explored. Notably, this technology was crucial for the recent approval of an siRNA drug targeting transthyretin (TTR) mRNA for treatment of Transthyretin-induced amyloidosis (hTTR) [61] and has played an even more significant role in the development of the recent emergency use authorized mRNA vaccines [62]. Such successes will surely pave the way for accelerated preclinical and clinical development of new RNA therapeutics. Other types of LNPs are being developed for use in clinical trials. For example, Wagner et al. developed 1,2-dioleoyl-sn-glycero-3-phosphatidylcholine (DOPC) nanoliposomal EphA2-targeting siRNA [63] to be administered intravenously, currently in clinical trials for treatment of patients with advanced malignant solid neoplasm (NCT01591356).

#### 2.3.3. Viral-Encoded AONs

The possibility of encoding AONs in viral vectors has attracted attention over the years [64,65]. In particular, recombinant adeno-associated viruses (AAVs) that remain episomal and do not integrate into the genome have received enormous attention. This technology has the potential of becoming part of the arsenal of enabling technologies for RNA therapeutics since an AAV-AON strategy could combine the best features of oligonucleotide therapeutics and gene therapies.

**Figure 2:**
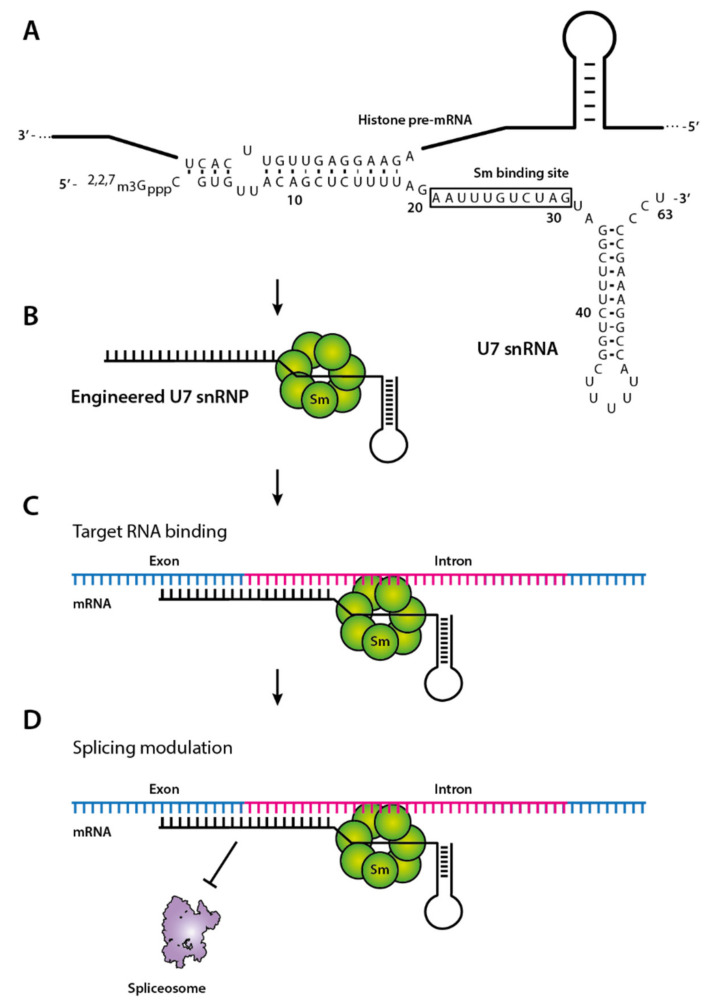
Modified U7 snRNP involved in splicing correction. (**A**) Sequence of U7 snRNA and histone pre-mRNA. The Sm binding site is indicated with a box. (**B**) Engineered U7 snRNA contains an optimized Sm, which is involved in splicing, resulting in binding of the spliceosomal Sm core proteins. The sequence of U7 snRNA, complementary to the histone downstream element, can be altered to the desired antisense sequence of the target gene. (**C**) The engineered U7 snRNP binds its target sequence by canonical base pairing and results in (**D**) modulation of the splicing due to steric blocking of the snRNP bound to the exon-intron junction.

The best-known system for delivering therapeutic oligonucleotides that can be packed in AAV is the U7 snRNA. This 60-mer small nuclear RNA (snRNA) normally forms a U7 snRNP particle and plays a critical role in processing histone pre-mRNA [66] (Figure 2A). The U7 snRNA consists of an antisense moiety targeting histone pre-mRNA, an Sm binding site, and a hairpin structure. It can be engineered by replacing the histone-specific antisense sequence with an AON sequence, acting as a steric blocker to induce splicing modulation [67] (Figure 2B–D). Furthermore, it can be encoded between viral inverted terminal repeats (ITR) and packed into AAV vectors. This AAV-AON strategy has successfully induced exon skipping in a mouse disease model [68]. There is currently a clinical trial (NCT04240314, run by Astellas Gene Therapies, formerly known as Audentes Therapeutics), testing this approach in Duchene Muscular Dystrophy (DMD) patients [69]. If successful, it will clear the way for new AAV-AON-based drugs.

## 3. Antisense Mechanisms: Gapmers, siRNAs, and Splice-Modulating Oligonucleotides

Since Paul Zamecnik pioneered the use of AONs [70,71], three main MoA with a direct therapeutic application have emerged: RNase H-mediated RNA degradation (gapmers), RNA interference (siRNAs), and splicing modulation (splice-modulating oligos). Here, we will review each technology’s biological concept and emphasize some innovations that will accelerate the development of new modalities.

### 3.1. RNase H-Mediated Degradation: Gapmers

Gapmers are AONs designed to recruit RNase H to degrade mRNA in a targeted fashion [72]. RNase H is a globally expressed endogenous endoribonuclease [73] capable of cleavage of a phosphodiester bond of an RNA molecule in the context of an RNA-DNA duplex [74]. The RNA-DNA duplexes can occur naturally in the cell. For example, to initiate DNA replication, RNA primers bind to DNA strands to form RNA-DNA duplexes. After initiation, the RNA primers are removed by the RNase H activity of DNA polymerase. There are two classes of RNase H enzymes: RNase H1, which is expressed in both nucleus and the cytoplasm, and RNase H2, which is more abundant than H1 but expressed only in the nucleus [75]. According to the crystal structure of human RNase H1 bound to a substrate RNA/DNA duplex [76], several acidic residues in the enzyme’s active site interact with phosphodiester bonds in the DNA strand (via Mg^2+^-coordination) located upstream of the actual cleavage site in the RNA strand.

**Figure 3:**
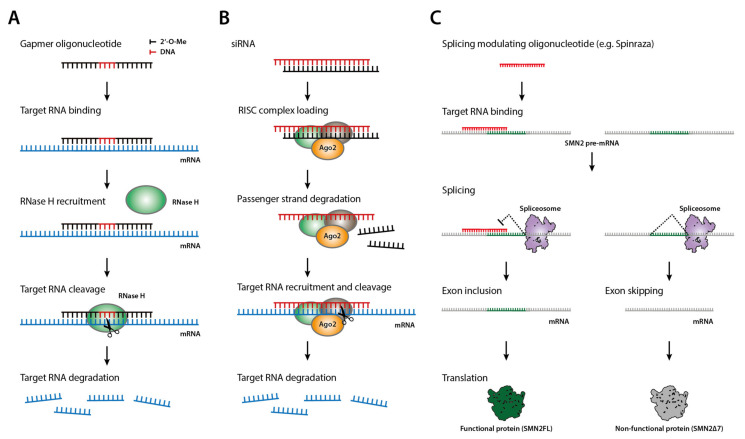
(**A**) Gapmer oligonucleotides contain a short DNA sequence embedded in a 2′-OMe-modified context. The gapmer binds its target mRNA, and this complex then recruits RNase H. The cleavage position of RNase H in the target mRNA is directed by the position of the DNA building blocks, and the cleaved mRNA is degraded. (**B**) Short interfering RNA (siRNA) contains two short RNAs with terminal overhangs, which recruit the RISC complex. This complex cleaves the non-targeting passenger strand RNA and then binds its target mRNA sequence-specifically. The target mRNA is then cleaved by the Argonaute protein and further degraded. (**C**) Splicing-modulating oligonucleotides bind their target RNA, often in proximity to an intron-exon junction, which results in the omission of this junction by the spliceosome during splicing. This can be used to correct pathogenicities caused by splicing defects, such as muscular dystrophy.

It was recognized decades ago that RNase H-mediated RNA degradation could be used as a therapeutic modality in the downregulation of genes [77,78]. It became more attractive when it was demonstrated that chimeric oligos (or gapmers) consisting of a central DNA-core of four deoxynucleotides flanked by stretches of 2′-O-methylated ribonucleotides, designed to base-pair with a target RNA of interest, could sufficiently elicit RNase-H mediated degradation of the RNA. While the design rules have been significantly improved over the years [79,80,81], the concept remains the same (Figure 3A). In a therapeutic setting, targeting a mutated mRNA could prevent the toxic effect associated with the expression of a gain-of-function or dominant-negative protein as a means of improving disease phenotypes. Once the gapmer hybridizes to the target mRNA, it can recruit the endogenous RNase H1, either in the nucleus or the cytoplasm, to digest the transcript.

During the early development of gapmers, it became clear that unmodified oligonucleotides were very unstable due to their susceptibility to nuclease-mediated degradation. Thus, to make gapmers more drug-like, developments in their chemistry would be required for successful therapeutic applications. The discovery of the phosphorothioate modifications as a means to prevent AONs from being degraded by endogenous nucleases [82] and increase their pharmacokinetic properties via better binding to serum proteins [83] was a landmark in this field.

Other types of modifications that occur at the sugar ring, namely 2′-OMe or 2′-MOE, as well as LNAs [84,85], also resulted in significant improvement of gapmers. Importantly, the chemical modifications developed and used over the years have improved the oligos’ pharmacokinetic properties without compromising target recognition by RNase H. Because the RNase H’s catalytic mechanism is not sequence-specific, gapmers are a flexible therapeutic tool.

In the last few years, three oligos, mipomersen, inotersen and volanesorsen, were approved for clinical use [86], all developed by Ionis Pharmaceuticals (San Diego, CA, USA) (Table 1). Mipomersen, a PS/2′-MOE/m^5^C modified gapmer targeting apo-B-100 mRNA, is used to treat familial hypercholesterolemia (FH) by reducing the plasma LDL-cholesterol levels [87]. Inotersen, a PS/2′-MOE 20-mer gapmer, was approved to treat hereditary Transthyretin Amyloidosis (hATTR), a fatal disease [88]. By degrading transthyretin mRNA via RNase-H degradation, it prevents the formation of deposits of transthyretin amyloid protein in the peripheral nervous system that would be toxic for the body. Volanesorsen, a PS/2′-MOE/m^5^C gapmer targeting apoC3 mRNA for treatment of Familial chylomicronaemia syndrome (FCS) (i.e., patients with high levels of blood triglycerides), was approved by the European regulatory authority (EMA) [89].

Several additional gapmers are in clinical development targeting liver, CNS, eye, muscle, and lung tissues, as well as tumor cells [90]. For instance, QR-1123 (formerly IONIS-RHO-2.5_Rx_) is currently in clinical trials conducted by ProQR Therapeutics (Leiden, The Netherlands) and was developed to treat autosomal dominant retinitis pigmentosa (adRP) caused by a c.68C>A mutation (P23H) in the rhodopsin gene (Table 1). QR-1123 is delivered via intravitreal injection. It was designed to inhibit the mutated version of the rhodopsin protein via a mutant allele-specific RNase-H-dependent knockdown mechanism to increase the wild-type rhodopsin function protein in photoreceptor cells present in the retina [91]. Another gapmer in clinical trials, IONIS-FB-L_RX_, was designed to reduce complement factor B to treat geographic atrophy associated with age-related macular degeneration (AMD) (Table 1). IONIS-FB-L_RX_, a GalNAc-conjugated 2′-MOE gapmer, targets the CFB gene in the liver following subcutaneous administration [92].

### 3.2. RNA Interference Mechanism: siRNAs

The RNA interference mechanism, discovered more than two decades ago [93], opens enormous possibilities for the silencing of genes as a means for the treatment of diseases. It was realized that duplex RNA complementary to a target gene could switch off its function and decrease both RNA and protein levels [94] and that the double-stranded RNA was processed into short 21–23 nt long oligos or small interfering RNAs (siRNA) [95] to fulfill the interference function. Dicer, an RNase III-type endonuclease, is responsible for processing the double-stranded RNA into the functional 21–23 siRNA duplexes, with overhangs in the 3′-end of each strand. The siRNA duplexes then bind to a protein known as Argonaute, which selects the antisense guide strand to the target RNA. The sense strand of the duplex, known as the passenger strand, is degraded. Together with the guide strand, the Argonaute forms an RNA-induced silencing complex (RISC) with the target RNA, ultimately cleaving it (Figure 3B). In a natural context, RISC uses naturally occurring microRNAs to recognize target mRNAs for down-regulation. Elbashiri et al. showed in 2001 that synthetic 21-nt duplexes of siRNAs could reliably inhibit endogenous expression of genes in mammalian cells in a very targeted manner [96].

The therapeutic potential for this MoA was immediately recognized as very significant, but the road towards clinical success was not straightforward. It took nearly two decades for the first siRNA drug to get approval to treat human diseases [61]. Multiple obstacles needed to be addressed. First, the sequence design of siRNA had to be optimized, and several rules had to be followed to generate more potent and target-specific siRNAs [97,98,99,100]. There were also other challenges common for any therapeutic antisense-based MoA, such as the RNA’s susceptibility to nuclease degradation or even the fact that exogenously introduced RNA can induce immune responses [101]. Besides, interference may produce off-target effects due to non-specific hybridization to similar RNA targets [102]. Due perhaps to a combination of these problems, some of the first siRNA clinical trials (bevasiranib and AGN211745, both for treatment of wet age-related macular degeneration) frustrated expectations for not meeting clinical endpoints [103]. However, another siRNA drug trial (CALAA-01), in which nanoparticles were used to pack and deliver an siRNA for cancer treatment, generated some hope [104]. It appeared that the nanoparticles were able to improve delivery efficiency and could also protect the siRNA when packaged inside the nanoparticles. Thus, to further improve the results, siRNA chemical modifications (to increase RNA stability, improve targeting, and escape immune response) appear to be desirable. As such, many different types of chemical modifications, including PS backbones, methylated 2′-O-alkyl moieties (2′-OMe and 2′-MOE), and 2′-F, were introduced into siRNAs. 2′-F was proven to improve on-target binding by increasing their binding affinity [19]. On the other hand, the 2′-OMe modifications have contributed to reducing siRNA duplexes’ immunogenicity [105]. The optimization of these chemical modifications has generated many lessons for future development in other MoA. Arguably, one of the most significant breakthroughs in the siRNA space was the use of GalNAc-siRNA conjugates, which showed high effectiveness in systemic administration for efficient delivery to the liver, specifically to hepatocytes [106]. In fact, almost one-third of the current siRNA in clinical trials are GalNAc-siRNA conjugates [107].

There are currently four siRNA approved drugs for the treatment of diseases such as hATTR amyloidosis with polyneuropathy (patisiran) [108], acute hepatic porphyria (givosiran) [109], primary hyperoxaluria type 1 (lumasiran) [110] and primary hypercholesterolemia (inclisiran) [111], all developed by Alnylam Pharmaceuticals (Cambridge, MA, USA) (Table 2).

Patisiran is administered intravenously as an LNP-based formulation that enhances the bioavailability, the cellular uptake and facilitates the endosomal escape of the siRNA drug [112]. It targets a conserved sequence in the 3′-untranslated region (3′-UTR) of both wild-type and mutant TTR transcripts. Patisiran siRNA is mostly unmodified, except that all pyrimidines in the sense strand and two uridines in the antisense strand are 2′-O-methylated. It also contains 2′-deoxythymidine dinucleotide overhangs at both 3′-ends (sense and antisense strands). The siRNA molecules are packaged with lipid nanoparticles which consist of four components: an ionizable cationic lipid to which the negatively charged siRNA binds, cholesterol, PEG-lipid, and distearolyphosphatidycholine (DSPC). The latter three components help to form the nanoparticle structure. This LNP technology was co-developed by Alnylam, Arbutus Biopharma and Acuitas Therapeutics (both based in Vancouver, BC, Canada), and the Cullis lab [113]. It not only is considered a critical milestone for the RNA Therapeutics field (patisiran was the first approved siRNA drug), but it has also paved the way for the quick development of Covid-19 mRNA vaccines, all of which are formulated in lipid nanoparticles [114]. One of the biggest challenges in the delivery of RNA drugs is the fact that once taken up by the cell, the nucleic acids (antisense oligos, siRNAs, or mRNAs) often get trapped in endosomes, failing to reach the target subcellular location (nucleus or cytoplasm, according to MoA) [115]. LNPs seem to not only protect therapeutic RNA from degradation but also improve the endosomal escape. Endosomes have an acidic interior that can protonate the ionizable component of LNPs and induce a structural change which ultimately disrupts the endosomes, releasing the RNA into the productive pathway [60].

Givosiran is a 2′ F, 2′-OMe, and partially PS-modified siRNA drug targeting aminolevulinate synthase 1 (ALAS1) mRNA in the liver. Instead of relying on the use of LNPs to target hepatocytes, givosiran is conjugated with a version of triantennary GalNAc. Aside from the fact that this conjugate improves the delivery of the siRNA in hepatocytes which express the ASGPR receptors [116], it also enables a slower release of the drug in tissues and permits subcutaneous administration, a more favored procedure than intravenous injections.

Lumasiran, a GalNAc-siRNA targeting glycolate oxidase (GO) mRNA is also administered via subcutaneous injections, as well as inclisiran, targeting PCSK9 transcripts. The chemistry of both siRNA drugs is relatively similar to that of givosiran [117].

The success of GalNAc-siRNA conjugates and LNP formulations is paving the way for future approvals of the multiple siRNA drugs currently in late-stage clinical development to treat diseases such as transthyretin-mediated (ATTR) amyloidosis, primary hyperoxaluria (PH) type 3 (PH3), hemophilia A and B, hyperoxaluria, acute kidney injury (AKI), and ocular pain and dry eye disease [118].

Micro RNAs (miRNAs) can similarly inhibit gene expression as siRNAs and have been described as potential gene silencing therapeutics in the form of microRNA mimics [119]. However, unlike siRNAs which target a single mRNA, miRNAs can target multiple transcripts, as their binding mechanism to mRNA is more imperfect than that of siRNAs, which ultimately is leading to a slower clinical development of this technology [120,121].

### 3.3. Splice-Modulating Oligonucleotides for Splicing Correction in Human Disease

Approximately 10% of human genetic diseases result from mutations that cause pre-mRNA splicing defects [122]. Pre-mRNA splicing occurs in the nucleus, producing mature mRNAs that are subsequently transported to the cytoplasm to direct protein synthesis [123]. During pre-mRNA splicing, non-coding intervening sequences (introns) are removed while the coding segments (exons) are joined together to form a strand of mature mRNA [124]. Splicing occurs in a large RNA-protein complex known as the spliceosome [125], where the important sequence elements within pre-mRNA are recognized. These sequence elements include the 5′ and 3′-splice sites, the branch site, and a number of regulatory sequences such as exonic/intronic splicing enhancers (ESE and ISE) and exonic/intronic splicing silencers (ESS and ISS). Recognition of these sequence elements occurs in a highly orchestrated manner, ensuring efficient and accurate splicing [126].

Although different variants (or isoforms) of mRNA (different arrangements of the exons) can be produced naturally through a process called alternative splicing, in some instances where genetic mutations occur in the aforementioned sequence elements, unwanted isoforms can also be generated, leading to diseases [127]. Often, these unwanted mRNA isoforms skip an exon (exon skipping) or include an intron (partial or total intron inclusion), resulting in the production of an altered protein product with no or altered function. In some instances, exon skipping, or intron inclusion can create a premature translation termination codon (PTC), leading to the activation of the nonsense-mediated mRNA decay (NMD) and the production of a truncated nonfunctional protein [128]. Even worse, mis-splicing due to genetic mutations may create an mRNA isoform with an altered open reading frame, thus yielding a different protein. Most disease-causing splicing mutations occur in the canonical 5′ and 3′ splice sites as well as the branch site, often via single-point substitutions. Examples of well-studied diseases caused by splicing mutations include retinitis pigmentosa (Usher syndrome) [129] and Leber’s congenital amaurosis [130].

It was suggested that small chemically modified RNA oligos could be efficiently designed to sterically block splicing factors and modulate splicing (Figure 3C). This would correct splicing defects and improve disease phenotypes [131]. This therapeutic MoA was first suggested by Dominski and Kole in 1993 [132] and has since established itself as an entirely new area in RNA therapeutics [133]. Because 5′ and 3′ splice sites and the branch site are relatively conserved sequences present in different introns, they are not ideal targets. Instead, it is common to target splicing enhancers either located in introns or exons (ISE or ESE, respectively). The first example of splicing modulation by oligonucleotides in humans was presented in 2009 by van Deutekom et al. In that study, patients with Duchenne’s muscular dystrophy, a severe degenerative genetic disease affecting the muscles, were given an intramuscular injection with a 2′-OMe/PS modified 18-mer AON (PRO051, later known as drisapersen) designed to skip exon 51 of the dystrophin gene [134]. However, the development of this AON drug by Biomarin Pharmaceutical (San Rafael, CA, USA) was eventually discontinued in late-stage (phase III) clinical trials due to the failure to meet primary clinical endpoints [135] (Table 3). In parallel, another company, Sarepta Therapeutics (Cambridge, MA, USA), was developing an AON (named exondys 51 or eteplirsen) with the same mode of action, that is, targeting the same exon of dystrophin (Table 3). Here, they used a completely different chemistry altogether: a PMO (morpholino) backbone. Unlike the more common negatively charged 2′-OMe/PS oligos, morpholino oligos have a neutral backbone that avoids nuclease degradation by cellular RNases. Eteplirsen was eventually approved for Duchenne patients by the FDA, but not by the European Medicines Agency (EMA) [136]. Golodirsen (or vyondys 53) [137] and casimersen (or Amondys 45) [138], two exon-skipping PMO-modified oligonucleotides, also developed by Sarepta Therapeutics, were recently approved by the FDA to treat DMD patients carrying mutations in exon 53 and 45 of the *DMD* gene, respectively (Table 3).

Another disease that benefited from novel splice-modulating oligonucleotide drugs is spinal muscular atrophy (SMA), a neuromuscular genetic disorder primarily caused by mutation/deletion of the *SMN1* gene, resulting in the production of a non-functional protein from this mutant gene. In humans, there also exists an SMN1 homologous gene called SMN2, the correct expression of which would fix this problem (SMN1 mutation/deletion). Unfortunately, however, a natural single nucleotide change in SMN2 (when compared with SMN1) leads to the skipping of exon 7, generating a non-functional or poorly functional SMN protein. To address this problem, the Krainer lab, in collaboration with Ionis Pharmaceuticals (USA), developed an m^5^C/2′-MOE/PS modified AON (named nusinersen, or spinraza) to reverse the disease phenotype by promoting the inclusion of exon 7 during the splicing of *SMN2* pre-mRNA [139,140] and succeeded [141] (Table 3). This is a remarkable success story in the RNA therapeutics field: in only ten years, research moved from a proof-of-concept in human cells [142] to phase III studies and FDA approval [143]. The trials for this AON were considered so successful that they were stopped early so that children treated with placebo could also receive the drug and benefit from it [144]. Recently this success story inspired an N-of-1 trial to treat neuronal ceroid lipofuscinosis 7 (CLN7) [145], also known as Batten disease.

Two splice-switching oligonucleotide drugs targeting two retinal disorders, Leber’s congenital Amaurosis 10 (LCA10) and Usher syndrome type II, sepofarsen (or QR-110) targeting CEP290 [43] and QR-421a targeting USH2A [146], respectively (developed by ProQR Therapeutics) are moving to late-stage clinical trials, after good clinical results in phase I/II studies [147] (Table 3).

**Table 3:** Splice-modulating AON approved or in clinical development.

Splice-Modulating Oligonucleotides					
Name	Company	Treatment for	mRNA Target	Status	Reference
Drisapersen	Biomarin	DMD	Dystrophin	Cancelled	[135]
Eteplirsen	Sarepta	DMD	Dystrophin	Approved	[136]
Golodirsen	Sarepta	DMD	Dystrophin	Approved	[137]
Casimersen	Sarepta	DMD	Dystrophin	Approved	[138]
Spinraza	Ionis	SMA	SMN2	Approved	[143]
Sepofarsen	ProQR	LCA10	CEP290	Phase II/III	[147]
QR-421a	ProQR	Usher syndrome type II	USH2A	Phase II/III	[146]

## 4. Therapeutic Potential of RNA Modifications

More than 170 nucleoside modifications have been described in different RNAs (snRNA, tRNA, rRNA, and mRNA) that can impact RNA structure and function and, ultimately, gene expression [10,148]. Three of the most common RNA modifications: 2′-O-methylation, inosine and pseudouridine, can be performed by RNA-guided mechanisms and are thus potentially useful for therapeutic application. Guide RNA oligonucleotides can be designed to have secondary structure features and/or chemical modifications that can harness endogenous RNA modification machinery in the cell. Here we will focus on these three RNA-guided RNA modifications.

### 4.1. 2′-O-Methylation: Artificial Box C/D snoRNAs

2′-O-methylation is a sugar ring modification that can occur at any nucleotide. This modification is highly abundant and widespread and is found in both non-coding RNAs (e.g., tRNA, rRNA, and snRNA) [149,150,151,152] and coding RNAs [153,154]. While this modification does not alter the hydrogen-bonding base-pairing, it affects the chemical and biophysical properties of the modified nucleotides and RNA. For instance, 2′-OMe groups favor the C3′-*endo* sugar conformation, which is a stabilizing effect in the context of RNA helices [155]. Further, 2′-O-methylation protects the RNA from nuclease degradation [156], thus prolonging the RNA’s half-life. Using NMR, Hala Assi et al. have shown that 2′-O-methylation results in increased thermal stability of the RNA [157]. 2′-O-methylation also increases the hydrophobicity of the RNA and reduces the reactivity of the sugar moiety.

**Figure 4:**
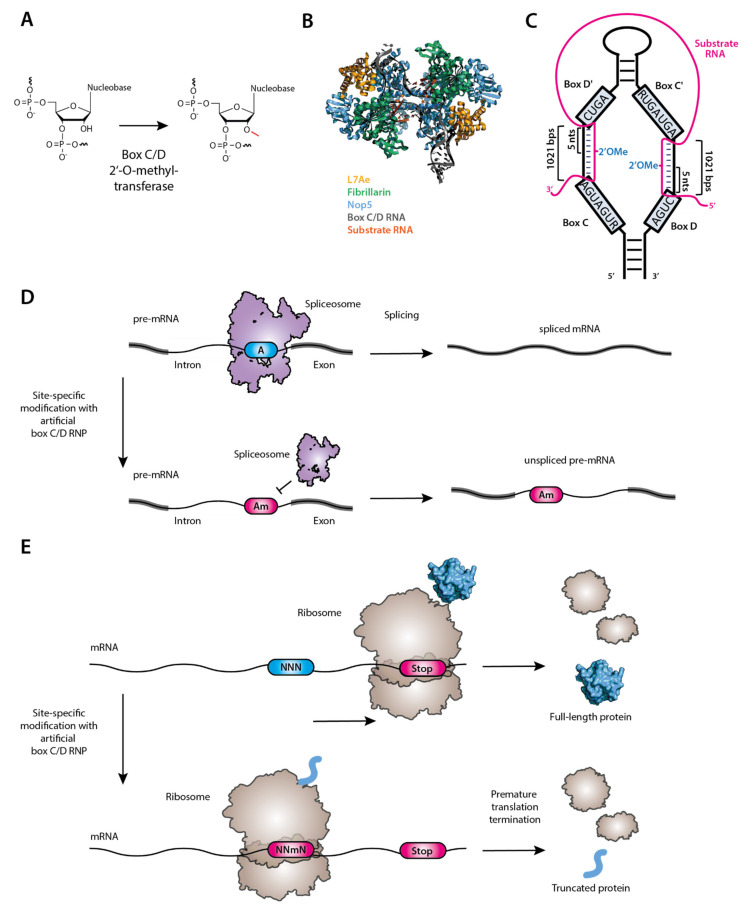
(**A**) Methyltransferase (standalone or box C/D RNP complex) catalyzes RNA 2′- O-methylation. (**B**) Crystal Structure of substrate-bound box C/D RNP (PDB: 5GIN [161]). (**C**) Schematic description of the secondary structure of the box C/D snoRNA (black). Substrate RNA is shown in magenta. (**D**) Site-specific, box C/D-directed methylation of the branchpoint adenosine (blue box) to yield Am (magenta box) results in inhibition of splicing of this intron. (**E**) Site-specific methylation of a central nucleotide within a sense codon results in premature translation termination, which can be used to inhibit the translation of nonfunctional, disease-relevant proteins.

2′-O-methylation (Figure 4A) is carried out by either standalone methyltransferases [158] or the box C/D ribonucleoproteins (RNP), each consisting of one small guide RNA (box C/D RNA) and four core proteins, Fibrillarin/Nop1 (the methyltransferase that transfers the methyl group to the 2′-OH), Nop56, Nop58 and Snu13 [159,160] (Figure 4B).

Despite their sequence differences, all box C/D RNAs fold into a unique secondary structure to provide the modification specificity through base pairing with its RNA substrate (Figure 4C) [162]. In the structure, there are two single-stranded sequences, one of which is sandwiched by box C (RUGAUGA, where R is purine) and box D’ (CUGA) motifs and the other by box C’ (RUGAUGA) and box D (CUGA). Both single-stranded sequences base-pair with their target RNA, specifying the two 2′-O-methylation residues, one of which is complementary to the nucleotide in the box C/D RNA that is located 5 nucleotides upstream of box D while the other is complementary to the box C/D RNA nucleotide located 5 nucleotides upstream of box D’ (box D/D’ + 5 rule) [160,163]. Taking advantage of the “box D/D’ + 5” rule, Zhao and Yu designed an artificial box C/D RNA to target the pre-mRNA branch point nucleotide (adenosine) for 2′-O-methylation, and by doing so, they showed that pre-mRNA splicing using that branch point adenosine was blocked [164,165] (Figure 4D). Applying this technology to target telomerase RNA, Huang and Yu successfully manipulated the telomerase activity [166], suggesting that this technology could potentially be an anticancer/antiaging therapy [167]. Recently, Elliot et al. has suggested that targeted methylation in a single nucleotide could be used to reduce or even inhibit translation [154], presumably because this RNA modification can disrupt codon reading and stall the translation elongation [168] (Figure 4E). Given that the target nucleotide is specified by the guide sequence, in theory, one should be able to construct designer box C/D RNAs with altered guide sequences to target any RNA for 2′-O-methylation at any desired site. As such, the designer box C/D RNA may be utilized as a potential therapeutic reagent. Many diseases such as cancer, asthma, and Alzheimer’s disease (AD) could be the potential targets of the targeted 2′-OMe molecular therapy [169].

### 4.2. Inosine: ADAR-Mediated A-to-I Editing

**Figure 5:**
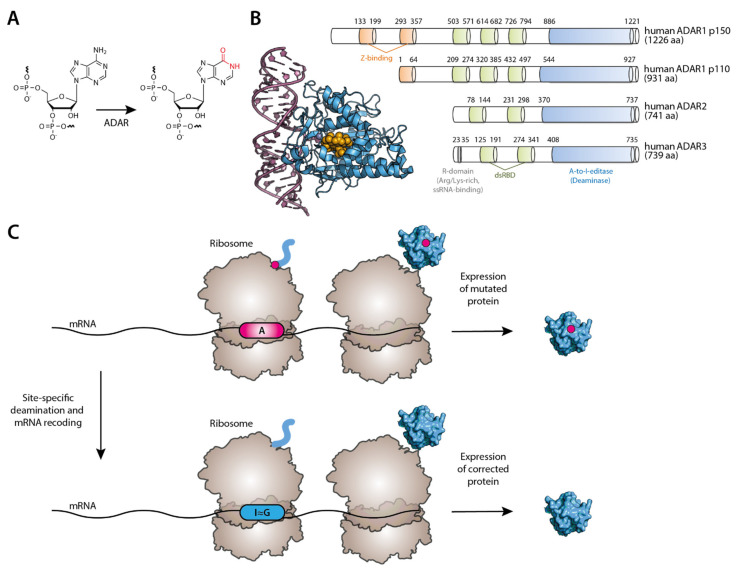
(**A**) ADAR-mediated RNA modification mechanism of deamination from adenosine to inosine. (**B**) Crystal structure of an RNA-bound ADAR fragment (PDB: 5ED1 [18]) and domain organization of human ADAR proteins. (**C**) Since inosine has a base-pairing behavior similar to guanosine, it is decoded as such by the ribosome. This allows for the correction of G-to-A point mutations to be reversed by site-specific deamination of target adenosines.

The inosine RNA modification has attracted much attention over the years [170,171,172]. It results from the hydrolytic deamination of adenosines in RNA (Figure 5A), in a process catalyzed by an enzyme called adenosine deaminases acting on RNA (ADAR). Inosines are recognized as guanosines by the translation machinery, and thus A-to-I editing results in an A-to-G conversion [173]. There are three types of ADAR enzymes: ADAR1, ADAR2, and ADAR 3. While ADAR1 and ADAR 2 are expressed in most tissues, ADAR3 is brain-specific and thought to be devoid of catalytic activity [174]. ADAR enzymes contain two main motifs: a double-stranded RNA binding domain (dsRBD) that can recognize duplex RNAs and a deaminase domain (DD) at the C-terminus that is responsible for the catalytic activity [175] (Figure 5B). The hydrolytic deamination mechanism involves base-flipping [176] of the adenosine base out of the double-stranded RNA, exposing it to the enzyme active site. There seems to be a preference for certain nucleotides flanking the target adenosine (uridine, cytosine, or adenosine at the 5′ of the target adenosine and a guanosine 3′ to it) [177,178]. Often, there is a cytidine residue as a mismatched base in the opposite RNA strand [179]. For ADAR1 p110 isoform and ADAR2, ADAR-mediated editing occurs mainly in the cell nucleus, although another interferon-inducible ADAR1 isoform (p150) can be localized in the cytoplasm and the nucleus [180].

The potential for inosines to recode transcripts and affect events such as splicing or translation has generated wide interest from the academic community and the biopharma industry [181] to correct disorders at the mRNA level, thus restoring or modulating protein function (Figure 5C). This is especially relevant to diseases resulting from G-to-A genomic single point mutations. One exciting discovery came to light about 26 years ago from the work of Wolf et al. They successfully used synthetic AONs complementary to the target RNA to site-specifically direct A-to-I conversion at a UAG premature stop codon (PTC) in dystrophin mRNA construct in Xenopus cell nuclear extracts and Xenopus embryos. However, some off-target events were detected in neighboring adenosines [182]. This prompted the need to develop more specific AONs not just to improve target specificity but also to recruit the ADAR enzyme to the editing site. The latter was suggested to be theoretically possible in a study by Jepson et al. [183]. Several researchers have tried to engineer ADAR enzymes covalently fused to or non-covalently bound to AONs to perform targeted RNA editing to tackle this issue further. Several different approaches have emerged using a SNAP protein [184], a λN-peptide [185], or a deactivated version of Cas13b [186]. While having their intricacies, these approaches relied on delivering artificial ADARs to the cells somehow [187]. Another strategy has also been pursued to deliver only the oligonucleotides (either chemically modified or genetically encoded) that would not only target a specific RNA site but would also be able to recruit the endogenous ADAR [taking advantage of ADAR’s double-stranded RNA Binding Domain (dsRBD)]. In particular, by attaching to the antisense moiety of the oligonucleotide a hairpin motif that mimics the R/G-motif of the glutamate receptor (GluR2) transcript [188], which binds to ADAR2, the Stafforst lab showed that trans-acting antisense guide RNAs could be engineered to perform the two critical functions (targeting the editing site and recruiting ADAR) [189]. The authors of this study showed that genetically encoded trans-acting guide RNA harboring a natural ADAR-binding sequence/structure could recruit endogenously expressed ADAR enzymes or transfected ADAR2 in human cells to recode a PTC in an eGFP construct into tryptophan. This approach was also successful in correcting a loss-of-function nonsense mutation in the PINK1 gene, which is linked to inheritable early-onset of Parkinson’s disease. The authors showed 35% of editing of the adenosine in a PINK1 PTC, in 293T cells co-expressed with the PTC-mutated PINK1 construct, the PTC-targeting guide RNA and ADAR2. In parallel, Fukuda et al. presented a similar strategy where the antisense region was linked to an ADAR-recruiting region [190] to repair a PTC artificially introduced in a GFP construct in HEK293 cells.

More recently, the Stafforst’s lab could execute this concept with chemically modified guide RNA oligos [191], suggesting that this delivery modality could be superior to plasmid-borne guide RNAs. Another delivery modality explored for ADAR-editing guide RNAs is the use of a viral vector, such as AAV, as proposed by the Mali’s lab [192]. Katreakar et al. tested this concept in two different mouse disease models by cloning the guide RNAs (with either an optimized R/G hairpin linked to the 5′ end of the guide sequence or two MS2 hairpins flanking the antisense guide RNA) and ADAR enzymes (wild-type ADAR1, ADAR2, or a hyper-editing version of ADAR: E488Q) in AAV vectors for correction of therapeutically relevant G-to-A mutations. Both intramuscular injection in Duchene mice (mdx) and systemic injection of mice suffering from ornithine transcarbamylase (OTC) deficiency (spfash mouse) resulted in targeted RNA editing and protein restoration. The fact that the mutant hyper-editing version of ADAR was responsible for a higher degree of off-target effects and even toxicity in the treated animals used in this study further underscores the importance of further developing the strategies to harness endogenous RNA editing machineries. In recent years, several biotech companies are taking the concept of ADAR-editing to preclinical and clinical development [181].

### 4.3. Pseudouridine: Artificial Box H/ACA Guide RNAs

**Figure 6:**
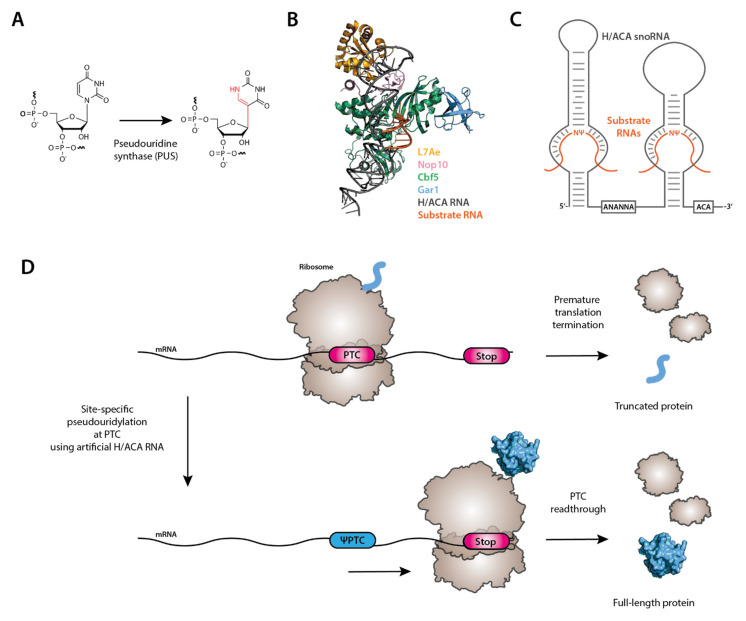
(**A**) Schematic of the conversion of U to Ψ catalyzed by standalone Pseudouridine synthase (PUS) or box H/ACA RNP. (**B**) Crystal structure of an archaeal substrate-bound full core box H/ACA snoRNP complex (PDB: 3HAY [200]). (**C**) Schematic description of the secondary structure of a eukaryotic box H/ACA snoRNA with substrate RNAs. (**D**) Site-specific pseudouridylation of stop codons results in ribosomal readthrough and allows for therapeutic correction of premature termination signals.

Pseudouridine (Ψ) is the most abundant modified nucleotide found in RNAs. Ψ is derived from uridine via pseudouridylation, a base-specific isomerization reaction, where the N1-C1′ is broken, and a new C5-C1′ bond is established (Figure 6A). Therefore, Ψ has chemical properties that are distinct from that of uridine. Specifically, the base of Ψ can rotate more freely due to the C5-C1′ bond (rather than the N1-C1′ bond in uridine). Further, because there is an additional -NH group in the base, Ψ contains an extra hydrogen bond donor group when compared with uridine. It has been demonstrated that Ψ can stabilize RNA structure by improving base stacking and favoring a C3′-*endo* conformation [193,194]. Ψ can be found in mRNA and in a number of different types of non-coding RNAs, such as rRNA, tRNA, and snRNA [195,196,197]. Pseudouridylation can be catalyzed by an RNA-guided mechanism involving box H/ACA snoRNPs. Box H/ACA RNPs each consist of one unique box H/ACA RNA and four core proteins, dyskerin/Cbf5/NAP57 (the pseudouridine synthase), Nop10, Gar1, and Nhp2 (L7Ae in Archaea) (Figure 6B). The RNA component folds into a conserved structure called the hairpin-hinge (box H)-hairpin-tail (box ACA) structure. In each of the two hairpins, there is an internal loop (known as pseudouridylation pocket) that serves as a guide to base-pair with its target RNA, thus specifying and positioning the target uridine at the base of the upper stem for modification by dyskerin [198,199] (Figure 6C). Clearly, target specificity is determined by base-pairing interactions between the guide sequence in the pseudouridylation pocket and the target sequence.

In a surprising finding, the Yu lab discovered a potential therapeutic application for this RNA-guided modification [201]. They showed that pseudouridylation of premature translation termination codons, caused by nonsense mutations, could be a potential nonsense suppression therapeutic strategy (Figure 6D). Nonsense mutations lead to mRNA degradation (due to the NMD surveillance pathway). A small fraction that escapes degradation is translated, but translation terminates prematurely at the PTC, resulting in the production of a truncated protein. Because all PTCs have a uridine at the first position, they could be recoded into sense codons using artificial box H/ACA snoRNAs designed to target the PTC uridine. The Yu lab presented this concept using a yeast reporter system expressing a reporter gene containing a PTC. Upon co-expression of an artificial box H/ACA snoRNA specific for the PTC present in the reporter mRNA, they observed full-length protein production. The authors were also able to identify the amino acids incorporated at the pseudouridylated PTC codons: primarily tyrosine and phenylalanine at ΨGA codons and serine and threonine at ΨAA and ΨAG codons. The Yu lab recently showed that the readthrough effect promoted by pseudouridylation of PTCs is independent of the target sequence, suggesting that this technology is theoretically applicable to all nonsense mutations [202]. Importantly, it appears that a single U-to-Ψ conversion at the PTC not only promotes PTC-readthrough during translation but also suppresses NMD. From the clinical perspective, effective suppression of NMD is also critically important given that in several diseases caused by nonsense mutations, there are extremely low amounts of transcript in the cell due to NMD. The low level of transcript poses a severe problem for drug treatments that attempt to correct the truncated protein only through promoting PTC-readthrough [203]. Therefore, inhibition of NMD is a stand-out feature of this technology with therapeutic impact [204,205].

Recently, Ψ was described to subtly alter the general decoding properties of pseudouridylated sense codons, suggesting that this RNA modification has additional recoding potential of the transcriptome [206]. It has also been reported that pseudouridylation of mRNAs leads to lower innate immune responses [207,208,209].

## 5. Conclusions

Antisense technology has come a long way since the early findings of Paul Zamecnik four decades ago and is now an established force in the biopharmaceutical industry. The advances in delivery (conjugates and LNPs), oligonucleotide design, and improvements in the chemical modifications have provided approved treatments for diseases with a high unmet medical need. Although only the most well-known MoA have so far succeeded in the clinics, a new generation of antisense-epitranscriptomic therapies is in the pipeline. The current momentum in the study of RNA modifications and the field of epitranscriptomics is sparking a strong interest in taking the lessons from antisense therapies to discover and develop new drugs based on novel MoA. The door is now open to further developments aimed to repair single point mutations while harnessing endogenous epitranscriptomics machinery.

The technologies developed and breakthroughs achieved for the antisense mechanisms, such as efficient large-scale manufacturing, next-generation chemical modifications, new conjugates that improve cellular uptake, and the revolution provided by LNP formulations, are now becoming enabling technologies for the new epitranscriptomic-based MoA. The development of new preclinical platforms, such as the use of patient-derived materials (e.g., organoids), as a complement or an alternative (when there is a lack of) to animal models holds great promise for validation of RNA editing modalities.

Alongside with CRISPR editing technologies [210,211,212,213,214], the established antisense technologies and the novel RNA modification-based MoA will play a significant role in biomedicine in this century.

## Figures and Tables

**Table 1:** RNase H gapmer AONs approved or in clinical development.

RNase H Gapmers					
Name	Company	Treatment for	mRNA Target	Status	Reference
Mipomersen	Ionis	FH	apo-B-100	Approved	[87]
Inotersen	Ionis	hATTR	Transthyretin	Approved	[88]
Volanesorsen	Ionis	FCS	apoC3	Approved	[89]
QR-1123	ProQR	adRP	Rhodopsin	Phase I/II	[91]
FB-LRX	Ionis	AMD	CFB	Phase II	[92]

**Table 2:** Approved siRNA drugs.

siRNAs					
Name	Company	Treatment for	mRNA Target	Status	Reference
Patisiran	Alnylam	hATTR	TTR	Approved	[108]
Givosiran	Alnylam	AHP	ALAS1	Approved	[109]
Lumasiran	Alnylam	Hyperoxaluria	GO	Approved	[110]
Inclisiran	Alnylam	Hypercholesterolemia	PCSK9	Approved	[111]

## Data Availability

Not applicable.
